# Genetic polymorphism of local Abkhazian grape cultivars

**DOI:** 10.18699/VJ21.092

**Published:** 2021-12

**Authors:** E.T. Ilnitskaya, M.V. Makarkina, I.V. Stepanov, I.I. Suprun, S.V. Tokmakov, V.Ch. Aiba, M.А. Avidzba, V.K. Kotlyar

**Affiliations:** North-Caucasian Federal Scientific Center of Horticulture, Viticulture, Winemaking, Krasnodar, Russia; North-Caucasian Federal Scientific Center of Horticulture, Viticulture, Winemaking, Krasnodar, Russia; North-Caucasian Federal Scientific Center of Horticulture, Viticulture, Winemaking, Krasnodar, Russia; North-Caucasian Federal Scientific Center of Horticulture, Viticulture, Winemaking, Krasnodar, Russia; North-Caucasian Federal Scientific Center of Horticulture, Viticulture, Winemaking, Krasnodar, Russia; Institute of Agriculture of the Academy of Sciences of Abkhazia, Sukhum, Abkhazia; Institute of Agriculture of the Academy of Sciences of Abkhazia, Sukhum, Abkhazia; North-Caucasian Federal Scientific Center of Horticulture, Viticulture, Winemaking, Krasnodar, Russia

**Keywords:** Vitis vinifera L., local grape varieties, genetic diversity, SSR-loci, Vitis vinifera L., местные сорта винограда, генетическое разнообразие, SSR-локусы

## Abstract

Local grape cultivars from different countries of the world are an important part of the gene pool of this culture. Of particular interest are the genotypes of the most ancient regions of viticulture. The territories of the subtropical zone of Georgia and the central part of Abkhazia belong to one of the centers of origin of the cultural grapevine. The purpose of the work was to genotype native Abkhazian grape cultivars, to study their genetic diversity based on DNA profiling data and to compare them with the genotypes of local varieties of other viticultural regions. Samples of plants were taken on the territory of the Republic of Abkhazia in private farmsteads and in the collection of the agricultural firm “Vina i Vody Abkhazii“ (“Wines and Waters of Abkhazia”). The genotyping of the Abkhazian cultivars Avasirhva, Agbizh, Azhapsh, Azhizhkvakva, Azhikvaca, Atvizh, Atyrkuazh, Achkykazh, Kachich was carried out using 14 DNA markers, 9 of which are standard microsatellite markers recommended for the identification of grape varieties. To improve our knowledge about the sizes of the identified alleles, we used the DNA of grape cultivars with a known allelic composition at the analyzed loci. Statistical analysis of the data showed that the observed heterozygosity for the analyzed loci exceeded expected values, which indicates a genetic polymorphism of the studied sample of varieties. Evaluation of genetic similarity within the analyzed group based on the results of genotyping at 14 loci showed that the cultivars Kachich and Azhapsh differed from the other Abkhazian varieties. The obtained DNA profiles of the Abkhazian cultivars were checked for compliance with DNA-fingerprints of grape varieties in the Vitis International Variety Catalogue. The Georgian varieties Azhizhkvakva and Tsitska turned out to be synonyms according to DNA profiles, two varieties from the Database (Italian Albana bianca and Georgian Ojaleshi) have differences in DNA-fingerprints from the varieties Atyrkuazh and Azhikvatsa only in one allele, respectively. When comparing the identified Abkhazian grape genotypes, their difference from the sample of Dagestan, Don, Greek, Turkish, Italian, Spanish, and French varieties and genetic similarity with the genotypes of Georgian grapes were shown.

## Abstract (ru)

Аборигенные сорта винограда различных стран мира – важная часть генофонда культуры. Особый интерес вызывают генотипы наиболее древних регионов виноградарства. Территории субтропической зоны Грузии и центральной части Абхазии относят к одному из центров возникновения культурной виноградной лозы. Целью работы было генотипирование аборигенных абхазских сортов винограда, изучение их генетиче- ского разнообразия на основе данных ДНК-профилирования и сравнение с генотипами местных сортов других регионов виноградарства. Образцы растений были отобраны на территории Республики Абхазия в частных подворьях и в коллекции агрофирмы «Вина и воды Абхазии». Генотипирование абхазских сортов Авасирхва, Агбиж, Ажапшь, Ажижкваква, Ажикваца, Атвижь, Атыркуажь, Ачкыкажь, Качич выполнено с помощью 14 ДНК-маркеров, девять из которых являются стандартными микросателлитными маркерами, рекомендованными для паспорти- зации сортов винограда. Для уточнения размеров идентифицированных аллелей в работе использовали ДНК сортов винограда с известным аллельным составом по анализируемым локусам. Статистический анализ данных показал, что фактическая гетерозиготность по анализируемым локусам превысила ожидаемую, что говорит о генетическом полиморфизме исследуемой выборки сортов. Оценка генетического сходства внутри анализируе- мой группы по результатам генотипирования по 14 локусам показала отличие сортов Качич и Ажапшь от осталь- ных абхазских сортов. Полученные ДНК-профили абхазских сортов были проверены на предмет соответствия ДНК-паспортам сортов винограда, представленных в международной базе данных. Грузинские сорта Ажижкваква и Цицка оказались синонимами по паспортам ДНК; у двух сортов из базы данных (итальянский Albana bianca и грузинский Оджалеши) обнаружены отличия в ДНК-паспортах от сортов Атыркуажь и Ажикваца соответствен- но только по одному аллелю. При сравнении идентифицированных абхазских генотипов винограда показано их отличие от выборки дагестанских, донских, греческих, турецких, итальянских, испанских и французских сортов и генетическое сходство с генотипами винограда Грузии.

## Introduction

The grapevines Vitis vinifera L. are cultivated by humans for already about 5000 years and it is the most economically important fruit crop in the world now. Local varieties from different regions of the world are an important part of the crop’s gene pool. Of particular interest are the genotypes of the most ancient viticulture regions. Western Transcaucasia and, especially, the subtropical zone of Georgia and the central part of Abkhazia are recognized as one of the centers of the origin of cultural grapevine. Many ancient local varieties as well as wild grapevines are found in these regions.

Currently, molecular genetic methods are widely used to assess the diversity of the gene pool of cultivated plants, including grape. Microsatellite (SSR) markers are most often used for these purposes. The standard set of 9 microsatellite loci has been developed for DNA fingerprinting of grape genotypes (Bowers et al., 1999; This et al., 2004; VIVC, 2021). The use of DNA markers in order to identify grape varieties and study their polymorphism made it possible to clarify many questions regarding homonym and synonym varieties, as well as to determine the most genetically similar and distant genotypes (Crespan, Milani, 2001; Fossati et al., 2001; Vokurka et al., 2003; Santiago et al., 2005; Moreno-Sanz et al., 2008; Cipriani et al., 2010; Goryslavets et al., 2015; Raimondi et al., 2015; Mandić et al., 2019; Papapetrou et al., 2020; Pastore et al., 2020). For example, using a genetic analysis of 35 autochthonous varieties of Bosnia and Herzegovina at 9 standard microsatellite loci, several synonyms and homonyms were identified. Comparison of genotypes from Bosnia and Herzegovina with Croatian grape varieties also revealed synonyms and homonyms among the two groups (Mandić et al., 2019).

PCR analysis of Crimean local varieties revealed that the Shabash, Manzhil al and Shabash krupnoyagodnyi varieties have identical DNA profiles. The variety Shabash krupnoyagodnyi is a clone of Shabash, and variety Manzhil al is a synonym of Shabash (Goryslavets et al., 2015).

178 grape varieties ranging from widely cultivated to nearly extinct, harvested in Emilia-Romagna (Northern Italy), were analyzed by 10 microsatellite markers (Pastore et al., 2020). The data obtained showed that, in this region, there are varieties cultivated in other regions of Italy and in other countries, but under different local names; however, unique genotypes were also identified. Of the 122 unique genotypes identified, 62 are not described in the literature, except for mentions in historical documents. They probably belong to the local gene pool and, possibly, are the autochthons of this region.

A collection of 1005 grape samples was genotyped at 34 microsatellite loci (SSR) in order to analyze genetic diversity and origins study (Cipriani et al., 2010). The comparison of molecular profiles revealed 200 groups of synonymies. The list was corrected taking into account full synonyms, which reduced the database to 745 unique genotypes.

Viticulture and winemaking are of particular importance for the people of Abkhazia. The antiquity of viticulture and winemaking of this region is evidenced by many archaeological and paleobotanical finds (Chamagua, 1968). The local type of grape culture even exists here: maglari – the cultivation of vines on trees. The autochthonous varieties of Abkhazia are characterized by a late ripening period and the ability to preserve the crop for a long time on the bushes. The fame of Abkhazian wines is mainly associated with the varieties Auasyrhua (Avasirhva), Kachich (Kachichi), Amlahu.

In the history of the viticulture of Abkhazia, there were periods due to political and economic reasons, when this industry either developed intensively, or fell into decline. The greatest damage was done when phylloxera appeared in the region: the plantings of local varieties were actively destroyed; significant areas were planted with a resistant American variety Isabella. Many varieties have been lost and are preserved in private farmsteads as single bushes. However, interest to local varieties is growing, and molecular genetic studies can be used for DNA profiling (fingerprinting) of local varieties, assessment of genotype polymorphism, and clarification of the origin of unknown cultivars

The purpose of the study was the genotyping of Abkhazian grape varieties and assessment of genetic diversity of the studied sample based on the polymorphism of microsatellite loci.

## Materials and methods

In this study, plants corresponding to the ampelographic descriptions of Abkhazian varieties given in the “Ampelography of the USSR…” were taken (Frolov-Bagrev, 1953, 1954; Negrul et al., 1963, 1970). Some samples of the varieties included in the research were collected from several geographical points of Abkhazia (Kachich, Avasirhva, Agbizh), others were taken from the collection of the agricultural company “Vina i Vody Abkhazii” (Atyrkuazh, Azhikvaca, Atvizh, Azhizhkvakva, Achkykazh, Azhapsh) (Table 1).

**Table 1.**
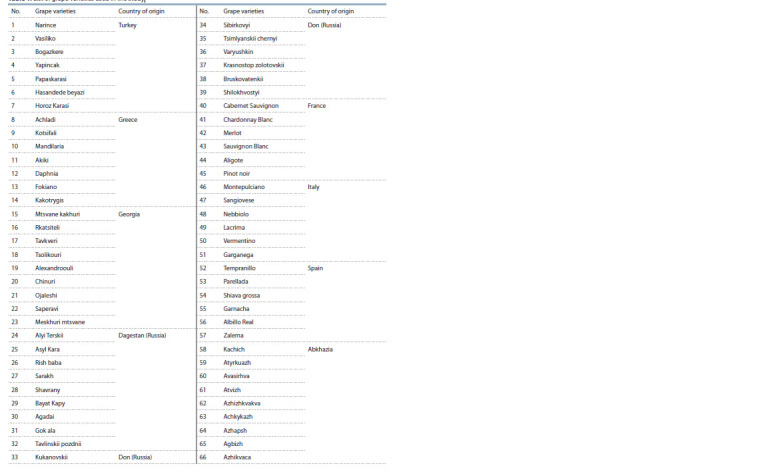
List of grape varieties used in the study

DNA was isolated from the crown leaves from annual shoots of three to five typical variety plants by the method using the CTAB buffer (Rogers, Bendich, 1985). A standard set of 9 microsatellite (SSR) markers recommended for identification of grapevine genotypes was used for DNA fingerprinting (Bowers et al., 1999; This et al., 2004; VIVC, 2021). For a more complete assessment of the studied sample’s polymorphism, 5 SSR markers were additionally included in the work (UDV737, GF09-46, ScORGF15-02, GF15-42, CenGen6) (Di Gaspero et al., 2012; Schwander et al., 2012; van Heerden et al., 2014; Zendler et al., 2017). DNA fingerprinting of the Azhizhkvakva, Kachich and Avasirhva varieties for 9 standard microsatellite loci was performed by us earlier (Ilnitskaya et al., 2019–2021).

PCR was carried out in a 20 μL reaction mixture containing 50 ng of genomic DNA, 1.5 units of Taq polymerase, 1× buffer for Taq polymerase with ammonium sulfate and magnesium, 2 mM MgCl2, 0.2 mM each dNTP (deoxynucleotide triphosphates) (SibEnzyme-M, Moscow) and 200 μM of each primer (Syntol, Moscow) using a BioRad device (USA), following these protocols: initial denaturation – 10 s at +95 °С; then 34 synthesis cycles: denaturation – 10 s at +95 °С, primer annealing – 30 s at +55 °С for markers VVS2, VVMD5, VVMD7, VVMD27, UDV737, CenGen6, at +58 °С – VrZAG62, VrZAG79, ScORGF15-02, GF15-42, and at +60 °С – VVMD25, VVMD28, VVMD32, GF09-46, elongation – 30 s at +72 °С; the final cycle (final elongation) – 3 min at +72 °С.

Separation and analysis of the PCR products lengths was carried out by capillary electrophoresis using ABI Prism 3130 genetic analyzer. The amplified fragments were aligned relative to the control (reference) genotypes that we included in the work, with a known allelic composition for the analyzed loci: Pinot noir (for markers VVS2, VVMD5, VVMD7, VVMD25, VVMD27, VVMD28, VVMD32, VrZAG62, VrZAG79), Saperavi severnyy (GF09-46), Seyve Villard 12- 375 (UDV737), Regent (ScORGF15-02, GF15-42, CenGen6).

Statistical processing of data on loci polymorphism in the studied sample was carried out using the program GenAlEx 6.5 (Peakall, Smouse, 2012). Genetic relationships were assessed with the PAST 2.17c program using the pairwise within-group unweighted mean (UPGMA) and principal coordinate methods (PCoA) (Hammer et al., 2001).

To study the genetic similarity of the autochthonous Abkhazian varieties with the grapevine’s local gene pool from other viticulture zones, we included a sample of varieties that are classified as aboriginal genotypes of Georgia, Greece, Dagestan, Don (Rostov region, Russian Federation), Spain, Italy, France, Turkey (see Table 1). These regions of viticulture also have an ancient history of V. vinifera L. cultivation, geographical proximity or historical ties with Abkhazia. The DNA profiles of local varieties genotypes for nine SSR loci, standard for genotyping V. vinifera, were taken by us from the international database Vitis International Variety Catalog (VIVC). Bayesian analysis was carried out in the Structure 2.3.4 program using 66 genotypes (see Table 1), the optimal number of clusters was 3, established using the Evanno method, calculation was carried out in the online program Structure Harvester (Evanno et al., 2005; Earl, vonHoldt, 2012).

## Results and discussion

The results of genotyping nine Abkhazian grape varieties (Kachich, Atyrkuazh, Avasirhva, Atvizh, Azhizhkvakva, Achkykazh, Azhapsh, Agbizh, Azhikvaca) by 14 microsatellite loci are presented in Table 2. The identified profiles of each variety for nine SSR loci (VVS2, VVMD5, VVMD7, VVMD25, VVMD27, VVMD28, VVMD32, VrZAG62, VrZAG79) were checked in the international database of grape varieties DNA fingerprints of Vitis International Variety Catalogue (VIVC, 2021). It was revealed that the allelic composition of the DNA profile of the Azhizhkvakva grape variety by nine microsatellite loci fully corresponds to the DNA profile of the Georgian aboriginal Tsitska grape variety, presented in the Database (Ilnitskaya et al., 2021). These varieties are similar in phenotypic characteristics and may be synonymous varieties or clone variations. The DNA profile of the Azhikvaca grape variety also showed a close similarity with the DNA profile of the Georgian aboriginal Ojaleshi grape variety: the difference was revealed only in one allele at VrZAG79 locus (VIVC, 2021).

**Table 2.**
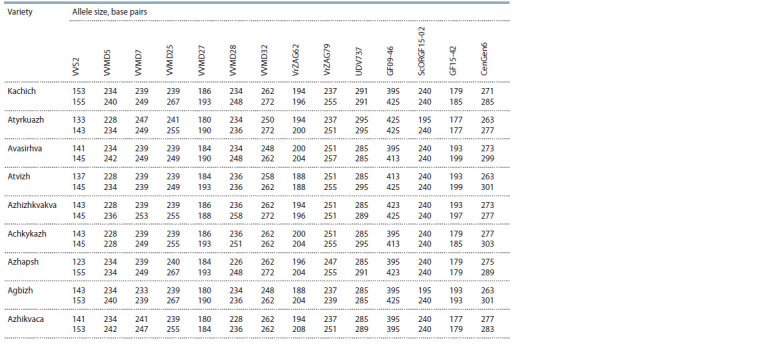
DNA prof iles of Abkhazian grape varieties

Since Georgia is the closest neighbor geographically and also a country with an ancient viticulture culture, the genetic similarity of native varieties of these regions is quite expected. However, we also revealed the similarity of the Atyrkuazh variety with a local Italian grape variety Albana bianca: the DNA profiles are identical except for one allele at the VrZAG79 locus (VIVC, 2021). Albana bianca is a relatively common variety and is found under other names in different countries (VIVC, 2021). The study of the ampelographic characteristics of ancient Italian variety Albana bianca, presented in the literature, shows a certain phenotypic similarity with variety Atyrkuazh. Due to the lack of DNA samples of the Albana bianca variety, it was not possible to compare genotypes for a larger number of SSR loci and clarify their level of genetic similarity.

In general, the genotypes of local Abkhazian varieties showed a fairly high polymorphism. The average value of observed heterozygosity (Ho = 0.810) exceeded the expected value (He = 0.712) in the studied sample of nine Abkhazian grape varieties at 14 microsatellite loci (Table 3). The least polymorphic locus was ScORGF15-02: only 2 alleles were detected. 11 alleles were identified on the most polymorphic locus CenGen6. It is worth noting that in the DNA profile of the Azhapsh grape variety at VVMD25 locus a very rare allele of 240 base pairs (bp) was identified, which was previously described in only one of the varieties presented in VIVC Database.

**Table 3.**
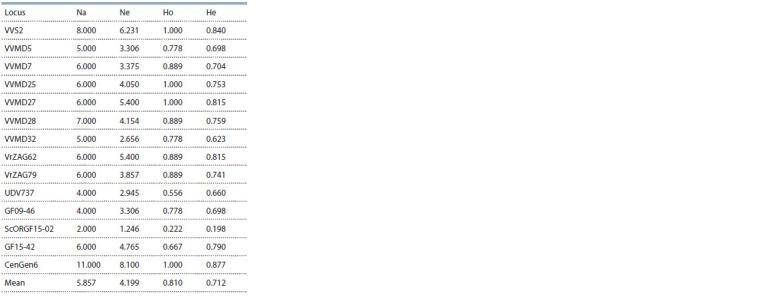
Characteristics of microsatellite loci in the studied sample of Abkhazian grape varieties

**Fig. 1.**
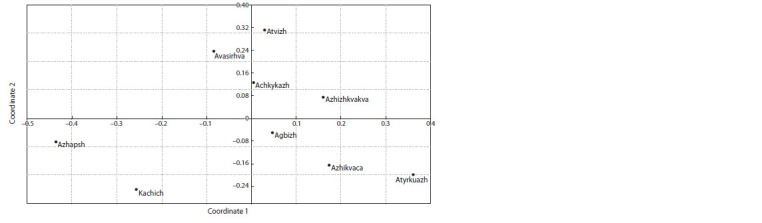
Dendrogram of genetic similarity of Abkhazian local grape varieties according to DNA prof iling data. Branching nodes show bootstrap percentage values calculated on the basis of 50,000 random samples.

**Fig. 2.**
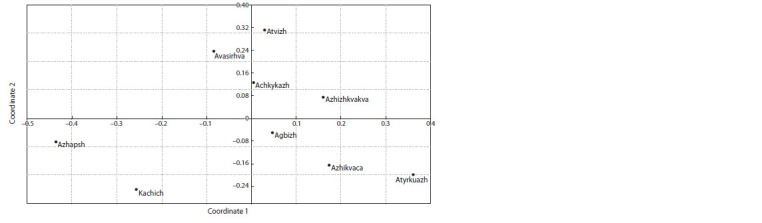
Distribution of Abkhazian grape varieties according to PCA analysis of DNA prof iling data.

To assess the genetic similarity of the studied Abkhazian varieties, a cluster analysis was carried out based on the data of SSR-genotyping. The analyzed varieties were divided into two clusters, one of which contains seven of the nine studied varieties and within which Agbizh, Atyrkuazh, Azhikvaсa were grouped into a separate subcluster, and Avasirhva, Atvizh, Achkykazh, Azhizhkvakva were grouped into another subcluster (Fig. 1). It is important to note that the genotypes of varieties Azhapsh and Kachich were allocated into one cluster. Localization of nine Abkhazian varieties in the PCA showed that the Azhapsh and Kachich varieties are grouped together and are located farther from the other seven varieties (Fig. 2).

**Fig. 3.**
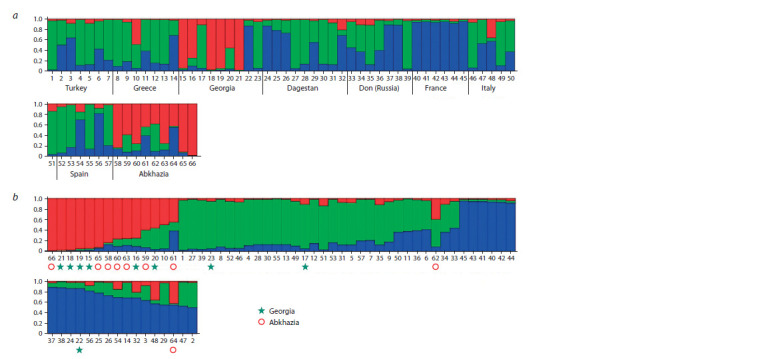
Bar plot of the results from Bayesian analysis on 66 grape varieties, genotyping with nine SSR markers: a, grouping of varieties by origin; b, clustering by genetic similarity. The vertical axis denotes the probability of assigning each genotype to the putative clusters, indicated by different colors. 1 – Narince, 2 – Vasiliko, 3 – Bogazkere, 4 – Yapincak, 5 – Papaskarasi, 6 – Hasandede beyazi, 7 – Horoz Karasi, 8 – Achladi, 9 – Kotsifali, 10 – Mandilaria, 11 – Akiki, 12 – Daphnia, 13 – Fokiano, 14 – Kakotrygis, 15 – Mtsvane kakhuri, 16 – Rkatsiteli, 17 – Tavkveri, 18 – Tsolikouri, 19 – Alexandroouli, 20 – Chinuri, 21 – Ojaleshi, 22 – Saperavi, 23 – Meskhuri mtsvane, 24 – Alyi Terskii, 25 – Asyl Kara, 26 – Rish baba, 27 – Sarakh, 28 – Shavrany, 29 – Bayat Kapy, 30 – Agadai, 31 – Gok ala, 32 – Tavlinskii pozdnii, 33 – Kukanovskii, 34 – Sibirkovyi, 35 – Tsimlyanskii Chernyi, 36 – Varyushkin, 37 – Krasnostop zolotovskii, 38 – Bruskovatenkii, 39 – Shilokhvostyi, 40 – Cabernet Sauvignon, 41 – Chardonnay Blanc, 42 – Merlot, 43 – Sauvignon Blanc, 44 – Aligote, 45 – Pinot noir, 46 – Montepulciano, 47 – Sangiovese, 48 – Nebbiolo, 49 – Lacrima, 50 – Vermentino, 51 – Garganega, 52 – Tempranillo, 53 – Parellada, 54 – Shiava Grossa, 55 – Garnacha, 56 – Albillo Real, 57 – Zalema, 58 – Kachich, 59 – Atyrkuazh, 60 – Avasirhva, 61 – Atvizh, 62 – Azhizhkvakva, 63 – Achkykazh, 64 – Azhapsh, 65 – Agbizh, 66 – Azhikvaca.

For a broader understanding of the genetic structure of the population of grape varieties in Abkhazia and the relationship with the world gene pool, they were compared with the genotypes of aboriginal varieties of other regions of viticulture (Georgia, Greece, Dagestan, Don (Rostov region of Russian Federation), Spain, Italy, France, Turkey). The DNA profiles of varieties on nine standard SSR loci were taken from the international VIVC Database. Bayesian analysis showed the greatest degree of similarity between Abkhazian varieties and Georgian grape varieties (Fig. 3). Moreover, among the group of Georgian varieties, three genotypes showed similarities with other groups of varieties (Tavkveri, Saperavi, Meskhuri mtsvane), while the group of Abkhazian varieties is more homogeneous (see Fig. 3, a).

Also, the group of French varieties taken in the study stands out for its uniformity and difference from others. In the group of Italian varieties, the genotype of the Nebbiolo variety is closest to the Abkhazian ones, it has many synonyms and is characterized by a late maturation period. Among the Greek varieties, we can note the Mandilaria variety genotype, which differs from the others in this group and is similar in structure to the varieties of Abkhazia. This may indicate the genetic relationships of the grape’s gene pool in Greece and Abkhazia. It is known that in the times of Ancient Greece, the territory of Abkhazia was ruled by Greece for a period of time. During this period, perhaps, the exchange of the gene pool of grapes could also have taken place.

Figure 3, b shows that most varieties of Georgia and Abkhazia form a single group and differ from other genotypes. Two varieties, Azhizhkvakva and Azhapsh, are outside this group, but it is also noticeable that the hypothetical population marked in red, which prevails in the group of Georgian and Abkhazian varieties, makes a significant contribution to the structures of these genotypes.

## Conclusion

Genotyping of local Abkhazian grape varieties Avasirhva, Agbizh, Azhapsh, Azhizhkvakva, Azhikvaca, Atvizh, Atyrkuazh, Achkykazh, Kachich was carried out using 14 DNA markers, including 9 generally accepted for DNA identification of grape varieties. The comparison of the identified DNA profiles on microsatellite loci VVS2, VVMD5, VVMD7, VVMD25, VVMD27, VVMD28, VVMD32, VrZAG62, VrZAG79 in the International Database shows the coincidence of the allelic compositions of the Azhizhkvakva cultivar with the Georgian local variety Tsitska, the Azhikvaca cultivar differs by one allele from the Georgian variety Ojaleshi. Also, the Atyrkuazh variety differs in one allele out of nine analyzed from the Italian grape variety Albana bianca.

The assessment of genetic structure of the population of grape varieties of Abkhazia and its relationship with the local gene pool of other regions of viticulture showed the similarity of Abkhazian varieties with Georgian and a difference from other groups of varieties of neighboring regions (Dagestan, Don, Turkey) and more remote regions of ancient viticulture (Greece, Italy, Spain and France). According to the results of the study, it is possible to assume that local varieties from the populations of the wild gene pool of grapevines are of autochthonous origin.

## Conflict of interest

The authors declare no conflict of interest.
